# New device for assessment of endothelial function: plethysmographic flow-mediated vasodilation (pFMD)

**DOI:** 10.1038/s41440-024-01770-z

**Published:** 2024-07-01

**Authors:** Shinji Kishimoto, Yu Hashimoto, Tatsuya Maruhashi, Masato Kajikawa, Aya Mizobuchi, Takahiro Harada, Takayuki Yamaji, Yukiko Nakano, Chikara Goto, Farina Mohamad Yusoff, Yoshitaka Iwanaga, Koshiro Kanaoka, Tomohiko Yada, Tomomasa Itarashiki, Yukihito Higashi

**Affiliations:** 1 Department of Regenerative Medicine, Division of Radiation Medical Science, Research Institute for Radiation Biology and Medicine, Hiroshima University, Hiroshima, Japan; 2 Department of Cardiovascular Medicine, Medical Corporation JR Hiroshima Hospital, Hiroshima, Japan; 3 Division of Regeneration and Medicine, Medical Center for Translational and Clinical Research, Hiroshima University Hospital, Hiroshima, Japan; 4 Center for Cause of Death Investigation Research, Graduate School of Biomedical and Health Sciences, Hiroshima University, Hiroshima, Japan; 5 Center for Radiation Disaster Medical Science, Research Institute for Radiation Biology and Medicine, Hiroshima University, Hiroshima, Japan; 6 Department of Cardiovascular Medicine, Graduate School of Biomedical and Health Sciences, Hiroshima University, Hiroshima, Japan; 7 Dpartment of Rehabilitation, Faculty of General Rehabilitation, Hiroshima International University, Hiroshima, Japan; 8 Department of Medical and Health Information Management, National Cerebral and Cardiovascular Center, Osaka, Japan; 9 Department of Cardiology, Sakurabashi Watanabe Hospital, Osaka, Japan; 10 Saraya Co. Ltd., Osaka, Japan

**Keywords:** Endothelial function, Flow-mediated vasodilation, Plethysmographic flow-mediated vasodilation

## Abstract

Measurement of flow-mediated vasodilation (FMD) in the brachial artery by using ultrasound is a well-established technique for evaluating endothelial function. To make the measurement quicker and simpler than the measurements of conventional ultrasound FMD (uFMD), we have developed a new noninvasive method, plethysmographic FMD (pFMD), to assess vascular response to reactive hyperemia in the brachial artery. The aim of this study was to determine the accuracy of measurement of pFMD in comparison to that of measurement of conventional uFMD. This study was a multi-center, cross-sectional study. We compared pFMD by a new device using cuff pressure and volume with conventional uFMD using ultrasound in 50 men (mean age, 41 ± 9 years). pFMD significantly correlated with conventional uFMD (β = 0.59, *P* < 0.001). In Bland–Altman plot analysis of pFMD and conventional uFMD, the mean difference of pFMD and conventional uFMD was 0.78%, and limits of agreement (mean difference ±2 standard deviations of the difference) ranged from −4.53% to 6.11%. We demonstrated validity of the new method for measurement of pFMD, which can automate the evaluation of endothelial function in a short time. Measurement of pFMD is simpler than measurement of conventional uFMD and may have reduced artificial bias compared to that of conventional uFMD measurement (URL for Clinical Trial: https://ethics.hiroshima-u.ac.jp/site/wp-content/uploads/2022/12/eki_giji20221213.pdf. Registration Number for Clinical Trial: E2022-0131).

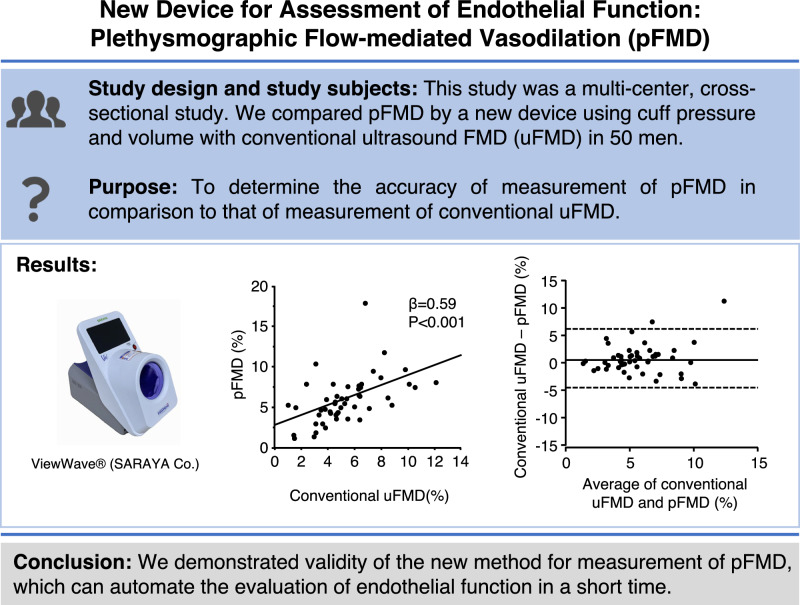

## Introduction

Endothelial dysfunction serves as an early stage in the development of atherosclerosis, ultimately leading to cardiovascular events [1, 2]. Endothelial function is assessed using strain-gauge plethysmography, in which a nitric oxide (NO) agonist or an NO antagonist such as acetylcholine, methacholine or bradykinin is intravascularly administered and subsequent blood flow in the forearm arteries is measured [3–6]. This method is regarded as the gold standard for evaluating endothelial function because it can yield specific insights into basal and stimulated NO release. However, measurement by strain-gauge plethysmography is difficult for clinical practice because it is an invasive and time-consuming procedure. Noninvasive assessment of endothelial function by measurement of flow-mediated vasodilation (FMD) in the brachial artery using ultrasound has been widely recognized and accepted in clinical practice [7–9]. Assessment of endothelial function through FMD independently predicts future cardiovascular events [10–13]. Furthermore, several treatments, including pharmaceutical treatment and lifestyle changes, have shown efficacy in improving endothelial function as assessed by FMD [14–18]. However, assessing ultrasound FMD (uFMD) necessitates a 5-minute forearm ischemia in the supine position, a component of the conventional uFMD measurement method that typically takes at least 15 min and demands specialized skills for vessel diameter measurement using ultrasound.

We have developed a new noninvasive method, plethysmographic FMD (pFMD), for assessing vascular response to reactive hyperemia in the brachial artery. This innovative approach relies on changes in vascular volume induced by ischemia, as measured through an automatic device using cuff pressure and volume. In this new method, a blood pressure cuff is placed around the upper arm and gradually deflated to 20 mmHg over a period of 30 s. The method of pFMD offers a less invasive and simpler means of assessing vascular function.

The aim of this study was to determine the accuracy of measurement of pFMD in comparison to that of measurement of conventional uFMD.

## Methods

This study was a multi-center, cross-sectional study. A total of 51 men volunteers aged 25–60 years were recruited at two centers (Hiroshima University Hospital and Saraya Co.) between July 2022 and March 2023. One of the 51 subjects was excluded because of poor-quality images obtained by conventional uFMD measurement. pFMD was measurable in all subjects. Finally, 50 subjects were enrolled in this study. Hypertension was defined as systolic blood pressure equal to or exceeding 140 mm Hg and/or diastolic blood pressure equal to or exceeding 90 mm Hg measured at least three times in a seated position [19]. Diabetes mellitus was defined according to the American Diabetes Association recommendation [20]. Dyslipidemia was defined according to the third report of the National Cholesterol Education Program [21]. The estimated glomerular filtration rate was calculated using the Japanese estimated glomerular filtration rate equation [22]. Subjects fasted the previous night for at least 12 h. The subjects were kept in the supine position or the sitting position in a quiet, dark, air-conditioned room (constant temperature of 22–25 °C) throughout the study. A 23-gauge polyethylene catheter was inserted into the left deep antecubital vein to obtain blood samples. Thirty minutes after maintaining the supine position, conventional uFMD was measured, followed by measurement of pFMD at least one hour later. pFMD was measured 30 min after maintaining a sitting position. The observers were blind to the subjects’ clinical status. All methods followed the Declaration of Helsinki and relevant guidelines and regulations. The Ethics Review Board of Hiroshima University approved the study protocol (Registration Number for Clinical Trial: E2022-0131). All participants in this study provided written informed consent before their involvement.

### Measurements of pFMD

The oscillometric method is often used for noninvasive blood pressure monitoring with a sphygmomanometer cuff tied around the upper arm. According to the theory, when the cuff pressure is equivalent to the arterial pressure, the arterial wall is assumed to be stress-free and the vessel experiences little distension [23, 24]. The cuff wave pressure is an indicator of the fluctuation in cuff pressure, which is caused by a change in the volume of the artery. The vascular response to reactive hyperemia in the brachial artery was assessed by cuff pressure variation and change in cuff volume of the pFMD. We assessed cuff pressure variation and change in cuff volume with a ViewWave (SARAYA, Osaka, Japan) (Supplementary Fig. 1). The cuff is inflated slowly to a level higher than 200 mmHg, and blood pressure is determined by analyzing oscillation signals obtained from the cuff pressure. This device requires a pre-set maximum cuff pressure. The maximum cuff pressure is set at 200 mmHg to achieve a systolic blood pressure of 50 mmHg or higher. We measured blood pressure in the upper arm in a resting sitting position during pressure elevation to 200 mmHg. The cuff pressure was maintained at 200 mmHg to cut off blood flow for 15 s, and then the cuff pressure was reduced to 20 mmHg for 30 s. Cuff pressure was maintained at 20 mmHg for 120 s, during which time changes in vascular volume were measured (Supplementary Fig. 2). In a preliminary study, there was a significant correlation between conventional uFMD, for which cuff pressure was maintained for 5 min, and short-time uFMD, for which cuff pressure was maintained for 40 s and then reduced for 30 s. Based on these results, pFMD also uses this protocol of reduced cuff pressure for 30 s. The cuff volume was measured based on the flow rate of gas supplied to and discharged from the cuff, as detected by the flow sensor. The cuff pressure-volume curve was used to create an approximate function F1 for the pressure-volume curve where the cuff pressure is higher than diastolic pressure (Supplementary Fig. 3). Arterial vessels are open when the cuff pressure is lower than diastolic pressure. Therefore, the pressure-volume curve will not approximate the approximate function F1 when the cuff pressure is lower than diastolic pressure. The difference between the cuff volume at the approximate function F1 when the cuff was 20 mmHg and the actual cuff volume at 20 mmHg is the volume that the arterial blood vessel opened, and this volume was defined as the baseline vessel volume. pFMD was calculated by the following equation: %pFMD = [(peak vessel volume − baseline vessel volume)/baseline vessel volume] × 1/2 × 100. The measurements were fully automated. Intra-coefficients were calculated from pFMD measured twice on different days under similar conditions, after 30 min while maintaining the sitting position. Intra-coefficients of variation for pFMD were 9.3% in our laboratory.

### Measurements of conventional uFMD

Vascular response to reactive hyperemia in the brachial artery was used to assess endothelium-dependent conventional uFMD. A high-resolution linear artery transducer was coupled to computer-assisted analysis software (MISTPILOT, SARAYA Co, Osaka, Japan) that used an automated edge detection system for the measurement of brachial artery diameter [25]. A blood pressure cuff was placed around the forearm. The brachial artery was scanned longitudinally 5–10 cm above the elbow. When the clearest B-mode image of the anterior and posterior intimal interfaces between the lumen and vessel wall was obtained, the transducer was held at the same point throughout the scan by a special probe holder to ensure consistency of the image. Depth and gain settings were set to optimize the images of the arterial lumen wall interface. When the tracking gate was placed on the intima, the artery diameter was manually tracked, and the waveform of diameter changes over the cardiac cycle was displayed in real-time using the conventional uFMD mode of the tracking system. This allowed the ultrasound images to be optimized at the start of the scan and the transducer position to be adjusted immediately for optimal tracking performance throughout the scan. The baseline longitudinal image of the artery was acquired for 30 s, and then the blood pressure cuff was inflated to 50 mm Hg above systolic pressure for 5 min. The longitudinal image of the artery was recorded continuously until 3 min after cuff deflation. Changes in brachial artery diameter were immediately expressed as percentage changes relative to the vessel diameter before cuff inflation. Conventional uFMD was automatically calculated as the percentage change in peak vessel diameter from the baseline value. The percentage of conventional uFMD [(Peak diameter − Baseline diameter)/Baseline diameter] was used for analysis. Inter- and intra-coefficients of variation for the brachial artery diameter were 3.5% and 2.6%, respectively, in our laboratory.

### Statistical analysis

Results are summarized as means ± SD for continuous variables and as percentages for categorical variables. Statistical significance was a probability value of <0.05. Relationships between variables were determined using Pearson’s correlation coefficients. Bland–Altman plot analysis was conducted to assess the level of agreement between pFMD and conventional uFMD [26]. To estimate the coefficient of correlation between pFMD and conventional uFMD, with a correlation coefficient equal to 0.4, it is necessary to sample size 47 subjects. The data were processed using JMP pro version 17 (SAS Institute. Cary, NC).

## Results

The baseline clinical characteristics of the subjects are summarized in Table 1. Of the 50 subjects, eight (16.0%) had hypertension, eight (16.0%) had dyslipidemia, one (2.0%) had diabetes mellitus, and 11 (22.0%) were current smokers. The mean values were 5.2 ± 2.4% for conventional uFMD and 6.0 ± 2.9% for pFMD.

**Table 1:** Clinical characteristics of the subjects

Variables	*n* = 50
Age, year	41 ± 9
Sex, men/women	50/0
Body mass index, kg/m^2^	22.9 ± 3.0
Systolic blood pressure, mmHg	121 ± 15
Diastolic blood pressure, mmHg	70 ± 12
Heart rate, bpm	63 ± 9
Total cholesterol, mg/dL	194 ± 31
Triglycerides, mg/dL	107 ± 99
High-density lipoprotein cholesterol, mg/dL	57 ± 13
Low-density lipoprotein cholesterol, mg/dL	116 ± 29
Glucose, mg/dL	97 ± 12
Hemoglobin A1c, %	5.5 ± 0.4
Blood urea nitrogen, mg/dL	14 ± 4
Creatinine, mg/dL	0.88 ± 0.11
Estimated glomerular filtration rate, mL/min/1.73 m^2^	78.4 ± 11.3
Current smoker, *n* (%)	11 (22.0)
Medical history, *n* (%)	
Hypertension	8 (16.0)
Dyslipidemia	8 (16.0)
Diabetes mellitus	1 (2.0)
Previous coronary heart disease	0 (0.0)
Previous stroke	0 (0.0)
Medication, *n* (%)	
Calcium channel blockers	4 (8.0)
Angiotensin-converting enzyme inhibitors/Angiotensin II receptor blockers	3 (6.0)
Beta-blockers	0 (0.0)
Lipid-lowering drugs	3 (6.0)
Antidiabetic drugs	1 (2.0)
Conventional ultrasound flow-mediated vasodilation, %	5.2 ± 2.4
Plethysmographic flow-mediated vasodilation, %	6.0 ± 2.9

Results are presented as means ± SD for continuous variables and percentages for categorical variables

### Relationships between pFMD and conventional uFMD

**Fig. 1:**
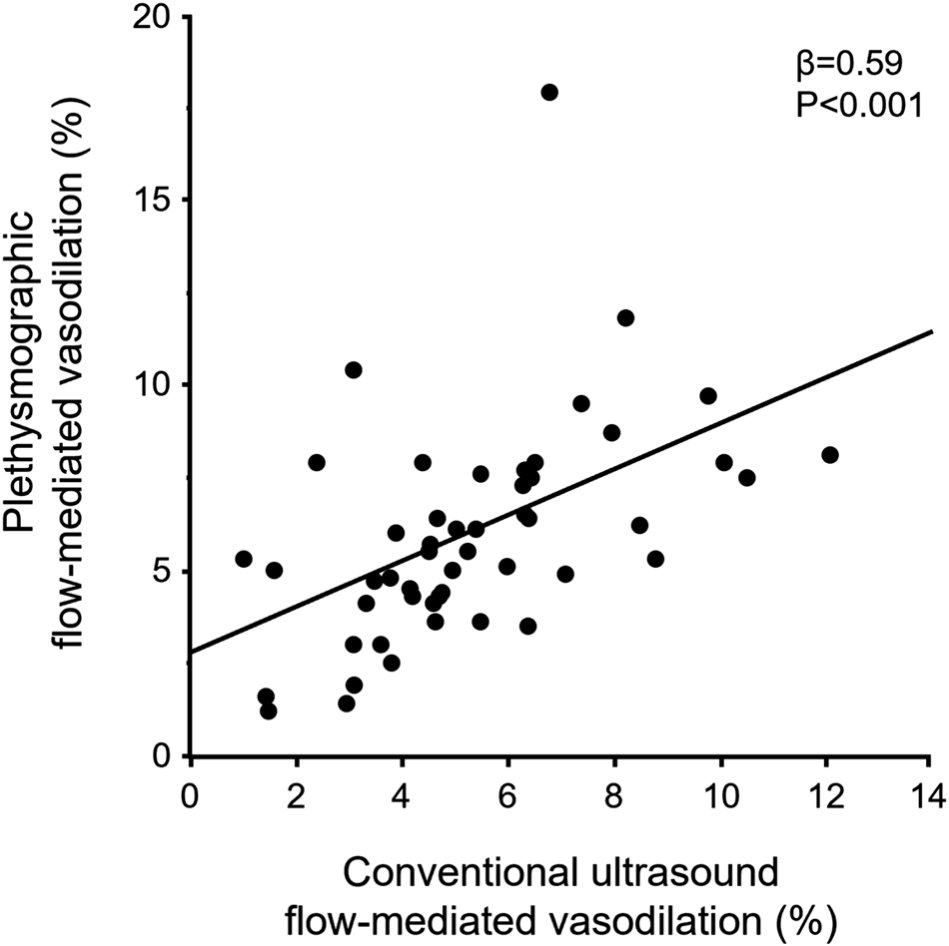
Scatter plot shows the relationship between plethysmographic flow-mediated vasodilation and conventional ultrasound flow-mediated vasodilation

**Fig. 2:**
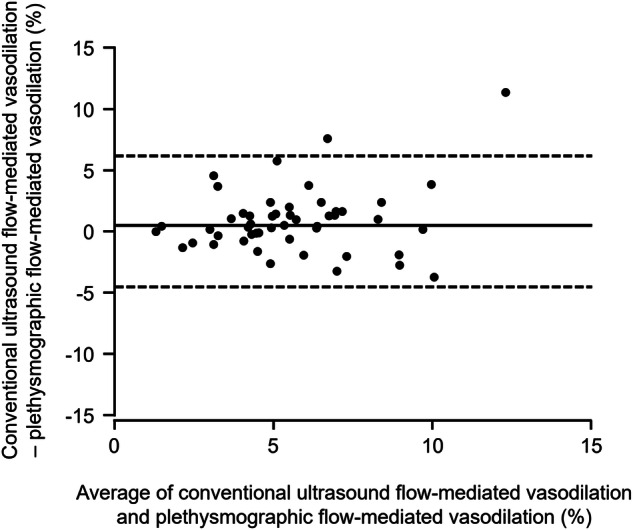
Bland–Altman plot of conventional ultrasound flow-mediated vasodilation and plethysmographic flow-mediated vasodilation. The bold line represents the mean difference between conventional ultrasound flow-mediated vasodilation and plethysmographic flow-mediated vasodilation. The dotted lines represent the mean difference ±2 standard deviations of the differences

pFMD significantly correlated with conventional uFMD (β = 0.59, *P* < 0.001) (Fig. 1). In Bland–Altman plot analysis of pFMD and conventional uFMD, the mean difference between pFMD and conventional uFMD was 0.78%, and limits of agreement (mean difference ±2 standard deviations of the difference) ranged from −4.53% to 6.11% (Fig. 2).

## Discussion

The present study demonstrated that pFMD significantly correlated with conventional uFMD. Bland–Altman plot analysis showed a high level of concordance between pFMD and conventional uFMD. It is easier, more convenient, and more comfortable for the subjects to measure pFMD with a shorter cuff inflation time in the sitting position.

Measurement of pFMD has several advantages as outlined below. Measurement of pFMD is simpler than measurement of conventional uFMD since fully automated measurements can be performed in the same way as blood pressure measurement and it requires only a short time for cuff inflation. Measurement of conventional uFMD with an ultrasound system requires specialized skills, as the operator must accurately position the transducer to image the brachial artery and manually or automatically monitor changes in brachial artery diameter [7, 27–29]. Measurement of pFMD requires only the application of a cuff on the upper arm, similar to measuring blood pressure. In addition, pFMD is measured in 5 min, including a cuff inflation time of 45 s. In contrast, measurement of conventional uFMD requires at least 15 min, including a cuff inflation time of 5 min and time to place the transducer in the proper position before cuff inflation. An incremental reduction in pressure release from the cuff results in optimal shear stress on the arterial wall, leading to subsequent dilation of blood vessels depending on the endothelium, despite a brief period of reduced blood flow in the upper arm. Therefore, measurement of pFMD has the benefit of avoiding artificial bias, such as manipulation of the transducer and operation of the ultrasound system, thereby enhancing the operation effectiveness of the present invention and no inter-measurer variation.

This study has some limitations. First, the correlations between pFMD and cardiovascular risk factors were not established due to validation only in males with low cardiovascular risk profiles. Further studies are needed to assess the relationships of pFMD with cardiovascular risk factors and future cardiovascular disease. Second, a difference exists between pFMD and conventional uFMD regarding the scope of arterial assessment. pFMD evaluates volume alterations in the arteries over the whole of the upper arm, including the brachial artery and small vessels of the upper arm, while conventional uFMD focuses only on a single brachial artery, which is classified as a macrovasculature. The relationship between pFMD and conventional uFMD has been established via the conversion of volume changes to diameter. Therefore, pFMD mainly evaluates the macrovascular endothelial function, although pFMD includes the evaluation of volume alteration in the arteries over the whole of the upper arm. pFMD might provide a more representative assessment of vascular endothelial function by integrating the effects of several arteries in the upper arm. Third, while the recommendations for measurement of FMD suggest using the supine position for the measurement of FMD, the measurement of pFMD is carried out in the sitting position. pFMD in the sitting position significantly correlated with conventional uFMD in the supine position. In our previous study, we showed that measurement of FMD in both the supine and sitting positions is appropriate for evaluation of endothelial function in various populations, including those with cardiovascular diseases [30, 31]. These studies suggest that endothelial function in different body positions may not change. It is possible that the same correlation was observed in the present study. Future studies should verify whether there is a difference in pFMD results between different body positions. Fourth, it is technically difficult to change the maximum cuff pressure settings for each patient, the maximum cuff pressure is set at 200 mmHg. In the present study, subjects had systolic blood pressure under 150 mmHg. Therefore, maximum cuff pressure was at least 50 mmHg higher than systolic blood pressure in all subjects. It will be necessary to confirm the correlation between pFMD and conventional uFMD when the cuff pressure is 200 mmHg in hypertensive patients with systolic blood pressure greater than 150 mmHg. Fifth, measurements of pFMD and conventional uFMD were performed on the same day. Therefore, we cannot deny the possibility that the vasodilation during the first uFMD measurement affected the results of the next pFMD measurement. However, Inaba et al. [32] have shown that uFMD fully recovered after 60 min following the initial uFMD. This result indicates that one hour is a sufficient interval for continuous FMD measurement. In the present study, at least one hour was allowed between the measurement of pFMD and that of conventional uFMD.

## Conclusion

We demonstrated the validity of a new method for measurement of pFMD, which can automate the evaluation of endothelial function in a short time. Measurement of pFMD is simpler than measurement of conventional uFMD and may have reduced artificial bias compared to that of conventional uFMD measurement. In further research, we should determine whether pFMD is a reliable predictor of cardiovascular outcomes and whether measurements of pFMD differ from measurements of conventional uFMD with respect to predicting cardiovascular outcomes.

## Supplementary information

Supplementary information

## Data Availability

The data presented in this study are available on request from the corresponding author.
