# Supplementary material for: New device for assessment of endothelial function: plethysmographic flow-mediated vasodilation (pFMD)
Source: Hypertens Res. 2024 Jul 1;47(9):2471–7. doi: 10.1038/s41440-024-01770-z (PMC11374665; doi:10.1038/s41440-024-01770-z)
Supplement: Supplementary file 1 — Supplementary information [file 41440_2024_1770_MOESM1_ESM.docx]

**Online Supplement**

**New Device for Assessment of Endothelial Function: Plethysmographic Flow-mediated Vasodilation (pFMD)**

Running title: plethysmographic flow-mediated vasodilation

Shinji Kishimoto, MD, PhD;^1^ Yu Hashimoto, MD;^2^ Tatsuya Maruhashi, MD, PhD;^1^ Masato Kajikawa, MD, PhD;^3^ Aya Mizobuchi, MS;^1^ Takahiro Harada, MD, PhD;^4^ Takayuki Yamaji, MD, PhD;^5^ Yukiko Nakano, MD, PhD;^6^ Chikara Goto, PhD;^7^ Farina Mohamad Yusoff, MD, PhD;^1^ Yoshitaka Iwanaga, MD, PhD;^8,9^ Koshiro Kanaoka, MD, PhD;^8^ Tomohiko Yada;^10^ Tomomasa Itarashiki, PhD;^10^ Yukihito Higashi, MD, PhD, FAHA^1,3^

^1^Department of Regenerative Medicine, Division of Radiation Medical Science, Research Institute for Radiation Biology and Medicine, Hiroshima University, Hiroshima, Japan

^2^Department of Cardiovascular Medicine, Medical Corporation JR Hiroshima Hospital, Hiroshima, Japan

^3^Division of Regeneration and Medicine, Medical Center for Translational and Clinical Research, Hiroshima University Hospital, Hiroshima, Japan

^4^Center for Cause of Death Investigation Research, Graduate School of Biomedical and Health Sciences, Hiroshima University, Hiroshima, Japan

^5^Center for Radiation Disaster Medical Science, Research Institute for Radiation Biology and Medicine, Hiroshima University, Hiroshima, Japan

^6^Department of Cardiovascular Medicine, Graduate School of Biomedical and Health Sciences, Hiroshima University, Hiroshima, Japan

^7^Dpartment of Rehabilitation, Faculty of General Rehabilitation, Hiroshima International University, Hiroshima, Japan

^8^Department of Medical and Health Information Management, National Cerebral and Cardiovascular Center, Osaka, Japan

^9^Department of Cardiology, Sakurabashi Watanabe Hospital, Osaka, Japan

^10^Saraya Co., Ltd., Osaka, Japan

Address for correspondence: Yukihito Higashi, MD, PhD, FAHA

Department of Regenerative Medicine, Division of Radiation Medical Science,

Research Institute for Radiation Biology and Medicine (RIRBM), Hiroshima University

1-2-3 Kasumi, Minami-ku, Hiroshima 734-8551, Japan

Phone: +81-82-257-5831 Fax: +81-82-257-5831

E-mail: yhigashi@hiroshima-u.ac.jp

**Methods**

**The theoretical relationship between pFMD and conventional uFMD**

The scheme of changes in vascular radius and vascular volume after cuff release is shown in supplemental Figure S4.

Pc indicates cuff pressure; ΔPc, change in cuff pressure; Vc, cuff volume; ΔVc, change in cuff volume; Vv, baseline vessel volume; R, vascular radius; ΔR, change in vascular radius; L, length of vessel; uFMD, ultrasound flow-mediated vasodilation; pFMD, plethysmographic flow-mediated vasodilation

Pc×Vc=constant=k

=(Pc+ΔPc)×(Vc-ΔVc)

= Pc×Vc +ΔPc×Vc- Pc×ΔVc-ΔPc×ΔVc

ΔPc×Vc- Pc×ΔVc-ΔPc×ΔVc=0

ΔPc×ΔVc/Pc×Vc is negligible since these are very small compared with other parameters.

ΔPc/ Pc-ΔVc/ Vc =0

ΔVc=ΔPc ×Vc/Pc

ΔVc/Vv=π((R＋ΔR)^2^−R^2^)L/πR^2^L

=(2RΔR+ΔR^2^)/R^2^

=(2R+ΔR) ΔR/R^2^

≒2RΔR/R^2^

=2ΔR/R

uFMD=100×2×ΔR/2×R

pFMD=100×2×ΔR/2×R

=100×ΔR/R

=100×ΔVc/2Vv

=100×(ΔPc ×Vc/Pc)/2Vv

=100×ΔPcVc/2VvPc

**Supplemental Figure 1**


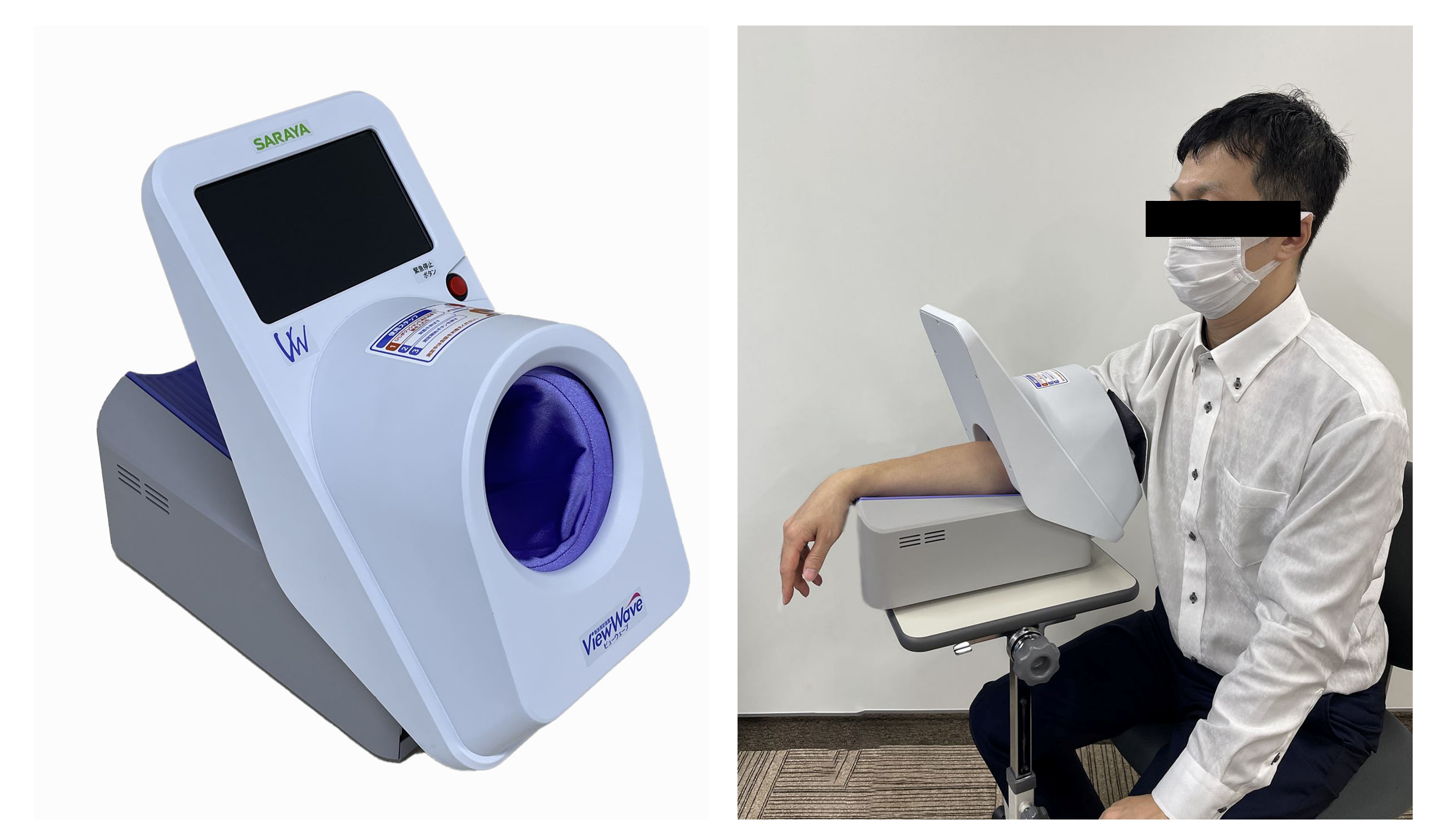


**Supplemental Figure 1 Legend:**

Device of measurement for plethysmographic flow-mediated vasodilation (pFMD)

**Supplemental Figure 2**

**
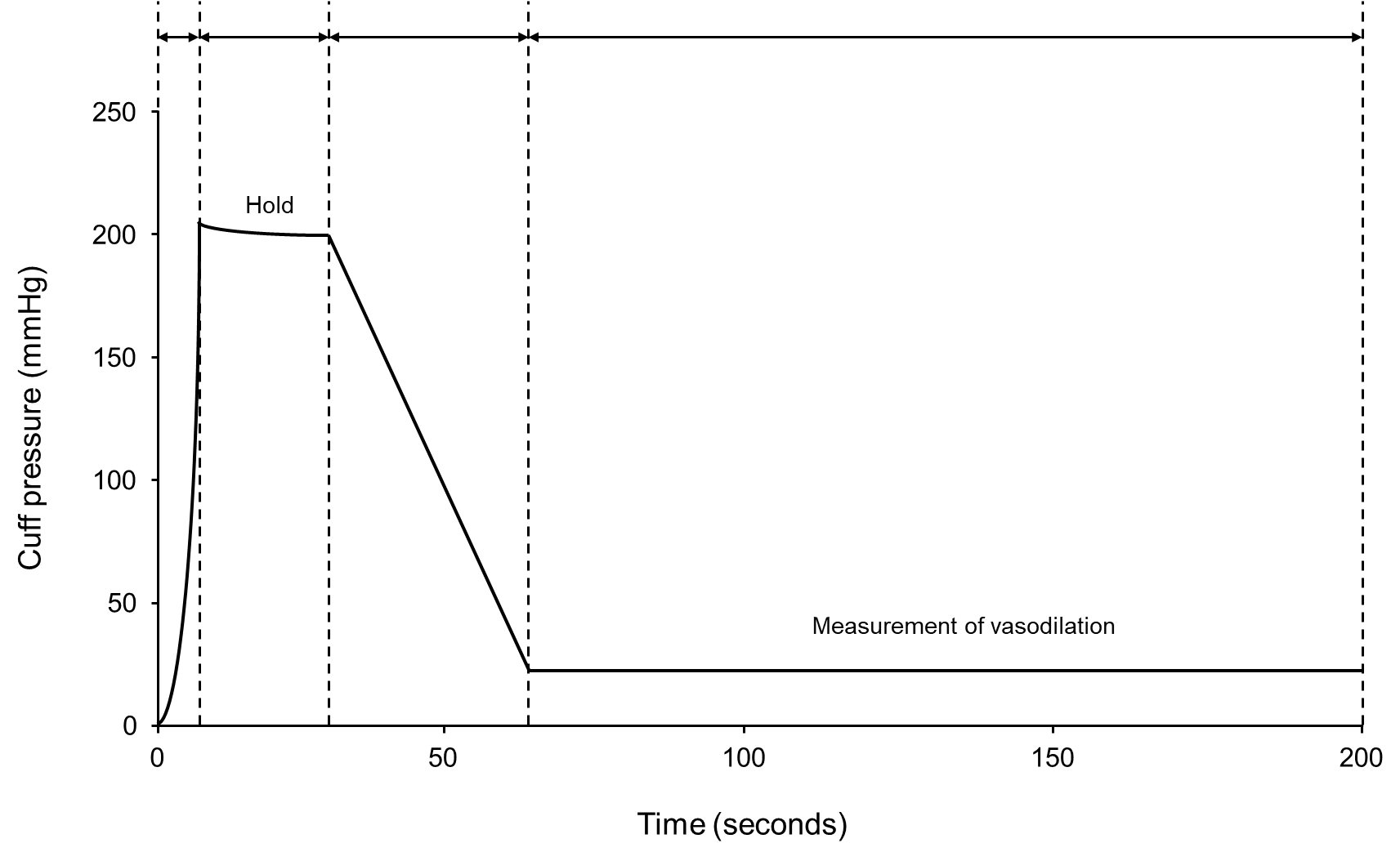
**

**Supplemental Figure 2 Legend:**

The relationship between cuff pressure and change over time

**Supplemental Figure 3**

**
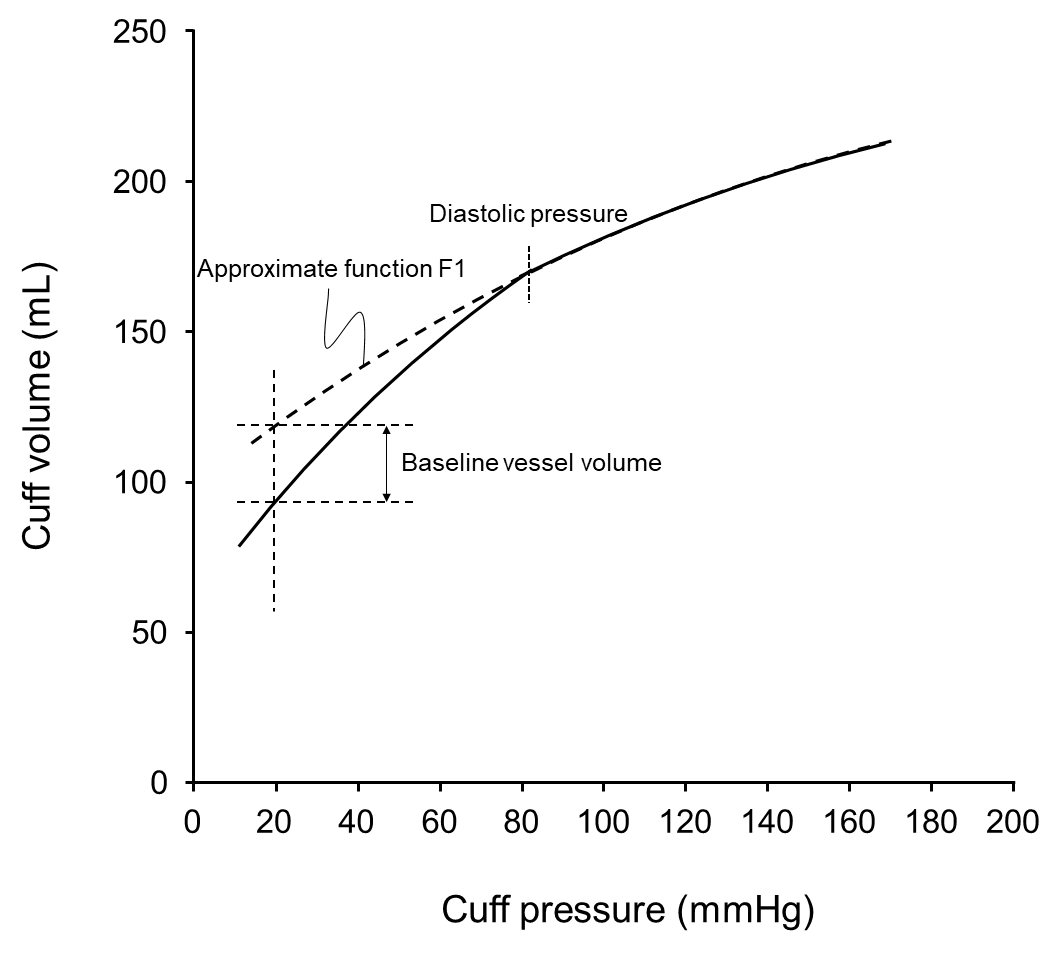
**

**Supplemental Figure 3 Legend:**

The relationship between cuff pressure and cuff volume and approximate function F1

**Supplemental Figure 4**


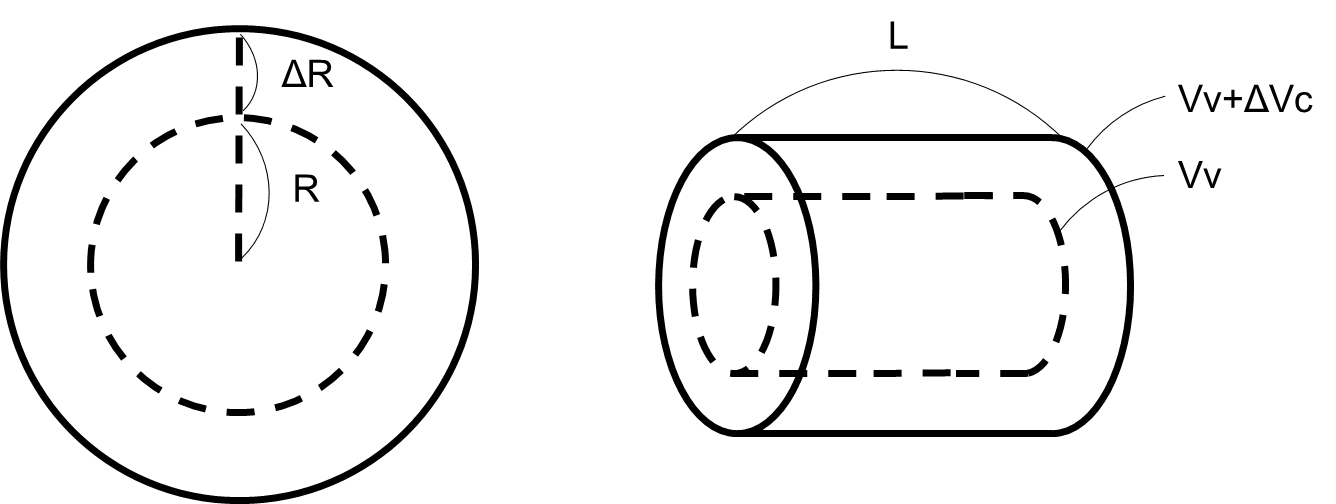


**Supplemental Figure 4 Legend:**

Changes in vascular radius after cuff release. R indicates vascular radius; ΔR, change in vascular radius; Vc, cuff volume; ΔVc, change in cuff volume; Vv, baseline vessel volume
